# Acute Aortic Dissection in Women: A Comprehensive Review of Sex-Specific Differences, Clinical Management, and Outcomes

**DOI:** 10.3390/jcdd13040158

**Published:** 2026-04-03

**Authors:** Vasiliki Androutsopoulou, Dimitrios E. Magouliotis, Andrew Xanthopoulos, Kalliopi Keramida, Metaxia Bareka, Konstantinos Stamoulis, Kosmas Tsakiridis, Thanos Athanasiou, John Skoularigis

**Affiliations:** 1 Department of Cardiothoracic Surgery, University Hospital of Larissa, 41110 Larissa, Greece; t.athanasiou@imperial.ac.uk; 2 Department of Cardiac Surgery Research, Lankenau Institute for Medical Research, Wynnewood, PA 19096, USA; magouliotisd@mlhs.org; 3 Department of Cardiology, University Hospital of Larissa, 41110 Larissa, Greece; anxanthopoulos@med.uth.gr; 4 Department of Cardiology, General Anti-Cancer Oncological Hospital, Agios Savvas, 11522 Athens, Greece; keramidakalliopi@hotmail.com; 5 Department of Anaesthesiology, University Hospital of Larissa, 41110 Larissa, Greece; bareka@uth.gr (M.B.); konstaarist@windowslive.com (K.S.); 6 Thoracic Surgery Department, “Interbalkan’’ European Medical Center, 55535 Thessaloniki, Greece; kosjohn@otenet.gr

**Keywords:** acute aortic dissection, sex, thoracic endovascular aortic repair, type A acute aortic dissection, type B acute aortic dissection

## Abstract

Acute aortic dissection (AAD) is a life-threatening cardiovascular emergency characterized by important sex-related differences in presentation, management, and outcomes. Although women account for a smaller proportion of cases, they typically present at older ages and more frequently exhibit atypical symptoms, hemodynamic instability, and complications such as pericardial effusion or tamponade, contributing to diagnostic delays and higher pre-hospital mortality. Beyond clinical factors, biological differences may influence disease expression in women. Menopause-associated vascular aging, hormonal modulation of extracellular matrix remodeling, and pregnancy-related hemodynamic and connective tissue changes may alter aortic wall integrity and susceptibility to dissection. Notably, women often experience dissection at smaller absolute aortic diameters, highlighting the potential importance of body-size indexing in risk stratification and surgical thresholds. In type A AAD, women are less likely to undergo extensive surgical repair in some cohorts, and although contemporary in-hospital mortality differences are narrowing, long-term survival disparities may persist. In type B AAD, women are more frequently managed conservatively, while outcomes following thoracic endovascular aortic repair appear broadly comparable between sexes. Pregnancy and the postpartum period represent particularly vulnerable windows, especially among patients with underlying heritable aortopathies. Greater awareness of sex-specific biological and clinical characteristics, incorporation of indexed aortic dimensions, and improved multidisciplinary management strategies are essential to optimize outcomes for women with acute aortic dissection.

## 1. Introduction

Acute aortic dissection (AAD) remains one of the most catastrophic cardiovascular emergencies, associated with substantial early mortality despite advances in diagnostic imaging, surgical techniques, and endovascular therapies [1,2]. Although men represent the majority of diagnosed cases, a growing body of evidence indicates that important sex-related differences exist in the epidemiology, presentation, and outcomes of AAD [2,3,4]. Women typically present at older ages and more frequently exhibit atypical or less specific symptoms compared with men, factors that may contribute to delays in diagnosis and treatment [2,3]. Registry data further suggest that women presenting with type A AAD are more likely to arrive in advanced clinical states, including hypotension, pericardial effusion, or cardiac tamponade, which are strongly associated with worse prognosis [2,4]. These differences highlight the need to better understand sex-specific determinants of disease expression rather than applying a uniform diagnostic and therapeutic approach to all patients.

Sex-related disparities in vascular disease extend beyond the aorta, with recent American Heart Association statements highlighting differences in presentation, diagnosis, and outcomes across the spectrum of peripheral and arterial vascular disease in women [5]. Women remain underrepresented in cardiovascular clinical trials, and many risk stratification models and therapeutic thresholds are derived predominantly from male-dominant cohorts [6]. Across cardiovascular conditions, women more frequently present with atypical symptoms and delayed recognition, and traditional risk prediction tools may inadequately capture sex-specific vulnerability. In addition, reproductive factors—including hypertensive disorders of pregnancy and adverse obstetric history—are increasingly recognized as cardiovascular risk enhancers [6]. Within this broader framework, AAD may represent a sex-modified vascular phenotype influenced by hormonal regulation, indexed biomechanical stress, and systemic vascular aging.

Beyond clinical presentation, biological mechanisms may contribute to variations in disease susceptibility and progression in women. Hormonal influences—particularly the decline in estrogen following menopause—have been associated with vascular stiffening, endothelial dysfunction, and alterations in extracellular matrix remodeling, processes that may compromise medial integrity and predispose to dissection [7,8]. Women have also been observed to experience AAD at smaller absolute aortic diameters, raising concerns that conventional surgical thresholds based on absolute size may underestimate risk when body size is not considered [7,9]. Pregnancy represents an additional unique risk state, characterized by increased blood volume, cardiac output, and hormonally mediated connective tissue remodeling, which together may increase vulnerability to dissection, particularly in patients with underlying heritable aortopathies [10,11,12,13,14]. These biological and biomechanical factors suggest that sex differences in AAD are not solely epidemiological observations but may reflect distinct pathophysiological pathways.

In addition to differences in disease biology, variations in management strategies have been reported. Women appear less likely in certain cohorts to undergo extensive surgical repair for type A dissection and may more frequently receive conservative management in selected type B scenarios [2,15,16,17]. Although contemporary improvements in perioperative care have narrowed in-hospital mortality differences in some series, higher pre-hospital mortality and potential long-term survival disparities remain areas of concern [2,3,4,15,18]. A comprehensive evaluation of sex-specific differences is therefore essential to refine diagnostic thresholds, optimize management strategies, and improve outcomes. This review synthesizes current evidence regarding epidemiology, biological mechanisms, clinical presentation, management patterns, and outcomes of acute aortic dissection in women. A central premise of this synthesis is that the tendency for women to dissect at smaller absolute aortic diameters, the so-called smaller diameter paradox, represents the most clinically consequential sex-specific finding in this field, one that directly challenges current risk stratification thresholds. Hormonal influences, menopause-associated vascular aging, and pregnancy-related connective tissue remodeling are discussed as the biological mechanisms that underlie and compound this phenomenon, rather than as independent parallel themes. Accordingly, this review adopts the ‘smaller diameter paradox’ as its central unifying construct, interpreting hormonal vascular aging, indexed biomechanical stress, and pregnancy-related connective tissue remodeling not as parallel themes but as mechanistic pillars of a single, sex-specific pathophysiological trajectory that spans from molecular biology to clinical outcomes.

## 2. Literature Search Strategy

This article represents a non-systematic narrative review of the published literature on sex-specific differences in acute aortic dissection. A comprehensive literature search was conducted across PubMed/MEDLINE, Embase, and the Cochrane Library, covering publications from January 2000 through February 2026. Seminal studies published prior to 2000 were included where judged to be of historical or foundational relevance. The primary search terms applied, individually and in combination, included: “acute aortic dissection,” “aortic dissection,” “sex differences,” “gender differences,” “women,” “female,” “pregnancy,” “postpartum,” “hormonal,” “menopause,” “type A aortic dissection,” “type B aortic dissection,” “thoracic endovascular aortic repair,” “TEVAR,” “heritable thoracic aortic disease,” and “risk stratification.” Priority was given to registry-based analyses, systematic reviews, meta-analyses, large observational cohort studies, and current clinical practice guidelines from the American College of Cardiology/American Heart Association and the European Society of Cardiology. Studies were included if they reported sex-stratified data on epidemiology, clinical presentation, pathobiology, management, or outcomes of acute aortic dissection. As a narrative review, formal PRISMA methodology was not applied; however, efforts were made to ensure balanced and comprehensive coverage of the available evidence, with particular attention to minimizing selection bias in the synthesis of registry-derived findings [2,3,4,7,8,9,15,19,20,21].

## 3. Epidemiology and Risk Profile of Acute Aortic Dissection in Women

Acute aortic dissection demonstrates a consistent male predominance across large registries [5]; however, women account for a substantial proportion of cases and represent a clinically distinct subgroup. Data from the International Registry of Acute Aortic Dissection (IRAD) indicate that women comprise approximately one-third of patients presenting with AAD and are, on average, significantly older at the time of diagnosis compared with men [2,3,4]. This age disparity is particularly notable in type A dissection and likely reflects both differences in underlying vascular biology and the delayed manifestation of degenerative aortopathy in women. The older age at presentation in women is consistently associated with a greater burden of comorbidities, including hypertension, which remains the most prevalent modifiable risk factor across both sexes [2,3]. Despite similar rates of hypertension, women are less frequently reported to have a history of prior aortic aneurysm diagnosis at the time of dissection, suggesting potential differences in surveillance patterns or disease recognition.

It is important to acknowledge, however, that much of the epidemiological evidence derives from registry-based analyses, most notably IRAD, which are subject to inherent selection and ascertainment bias. Enrollment is limited to participating tertiary centers, potentially underrepresenting community-based presentations and pre-hospital deaths—events that disproportionately affect women and may therefore lead to underestimation of the true sex-specific burden of disease [2,3,4]. Furthermore, observed differences in age, comorbidity profile, and presentation severity between sexes may partly reflect confounding rather than independent biological effects, as most registry analyses do not perform multivariable adjustment incorporating sex-specific interaction terms.

Beyond traditional cardiovascular risk factors, sex-specific differences in heritable and connective tissue disorders warrant consideration. While conditions such as Marfan syndrome, Loeys–Dietz syndrome, and vascular Ehlers–Danlos syndrome affect both sexes, pregnancy-related hemodynamic stress may unmask underlying aortopathy in women with genetically mediated connective tissue vulnerability [10,11,12,13,14]. Turner syndrome, uniquely affecting women, further exemplifies the importance of sex-linked genetic risk in aortic disease. In this context, systematic assessment of family history and consideration of genetic evaluation are clinically relevant, as identification of heritable thoracic aortic disease (HTAD) directly influences surveillance intensity, prophylactic intervention thresholds, and reproductive counselling [19,20]. In addition, observational data suggest that women may experience type A dissection at smaller absolute aortic diameters compared with men, though this pattern has been less consistently characterized in type B disease [7,9], raising the possibility that body surface area–indexed measurements provide a more accurate assessment of rupture or dissection risk [5]. Collectively, these findings challenge the reliance on absolute diameter thresholds alone and support a more individualized, sex-aware approach to risk stratification.

Pregnancy constitutes a distinct epidemiologic subset of AAD, with cases clustering during the third trimester and early postpartum period, when hemodynamic load and hormonally mediated vascular remodeling are most pronounced [10,11,12,13,14]. Although pregnancy-associated dissections are rare in absolute terms, they account for a disproportionate share of dissections in younger women and are often linked to underlying heritable thoracic aortic disease. Importantly, epidemiologic patterns suggest that women without previously recognized aortic dilation may still experience dissection in this context, underscoring the interplay between genetic predisposition, vascular remodeling, and acute physiological stress. Taken together, these epidemiological observations indicate that risk assessment in women must integrate age-related vascular changes, genetic susceptibility, pregnancy status, and indexed aortic dimensions rather than relying solely on traditional models derived largely from male populations.

These epidemiological observations carry direct clinical implications that extend beyond demographic description. The consistently older age at presentation in women, combined with a lower rate of pre-existing thoracic aortic aneurysm diagnosis [2,3], suggests that a substantial proportion of women reach the point of dissection without prior identification of aortic pathology, thus reflecting gaps in surveillance rather than a true absence of prodromal disease. The higher prevalence of atypical or non-specific symptoms in women increases the likelihood of misclassification in emergency settings, contributing to delays in definitive cross-sectional imaging and, ultimately, to the higher rates of pre-hospital mortality and advanced hemodynamic compromise observed at presentation [2,3,15]. Critically, the epidemiological finding that women dissect at smaller absolute aortic diameters [7,9] is not an isolated observation but the clinical expression of the biological mechanisms detailed in the following section, hormonal vascular aging, indexed biomechanical stress, and pregnancy-related remodeling, which together define a distinct and underrecognized risk trajectory in women.

## 4. Sex-Specific Pathobiology and Mechanistic Considerations

Sex-related differences in acute aortic dissection are unlikely to be explained solely by epidemiological patterns or age distribution. Instead, they appear to reflect fundamental differences in vascular biology, hormonal regulation, extracellular matrix composition, and biomechanical adaptation, all of which converge on a single clinically critical observation: women frequently dissect at smaller absolute aortic diameters than men, a phenomenon that challenges the validity of fixed diameter thresholds in female risk stratification. Understanding the biological mechanisms that drive this disparity is therefore central to contextualizing why women present later in life and may exhibit distinct patterns of disease progression.

Estrogen plays a central role in vascular homeostasis. Through modulation of endothelial nitric oxide synthase activity, estrogen enhances nitric oxide bioavailability, contributing to vasodilation and protection against endothelial dysfunction. In addition, estrogen exerts anti-inflammatory effects and regulates extracellular matrix turnover by influencing matrix metalloproteinase activity and collagen synthesis. The decline in estrogen levels following menopause is associated with increased arterial stiffness, impaired endothelial function, and alterations in collagen–elastin balance within the aortic wall [7,8,9]. These changes may promote medial degeneration, reduce adaptive compliance, and increase susceptibility to intimal tear formation under hemodynamic stress. The later age at presentation of AAD in women likely reflects the cumulative effects of vascular aging and hormonal withdrawal, suggesting that menopause represents a biologically meaningful inflection point in aortic vulnerability.

Beyond hormonal modulation, structural and biomechanical differences may alter dissection thresholds. Women generally have smaller aortic diameters and lower absolute wall tension compared with men; however, when indexed to body surface area, the relative degree of dilation at the time of dissection may be comparable or even greater [7,9]. According to Laplace’s law, wall stress is proportional to vessel radius and intraluminal pressure; therefore, reliance on absolute diameter thresholds may underestimate risk in smaller individuals. The observation that women frequently dissect at smaller absolute diameters, the so-called “smaller diameter paradox”, raises important questions regarding current guideline criteria for prophylactic surgery [19]. Indexed aortic measurements may better capture true biomechanical stress and improve risk stratification in women [7,9].

Extracellular matrix remodeling further contributes to sex-specific vulnerability. Medial degeneration in thoracic aortic disease is characterized by elastin fragmentation, smooth muscle cell loss, and increased matrix metalloproteinase activity. Hormonal influences may modulate these processes, particularly during periods of endocrine fluctuation such as menopause and pregnancy [7,8]. Differences in collagen cross-linking and connective tissue composition between sexes may influence both tensile strength and compliance of the aortic wall, potentially altering patterns of dissection propagation or intramural hematoma formation [2,3,4]. While direct mechanistic human data remain limited, translational evidence supports the concept that sex hormones influence vascular remodeling pathways in ways that may affect susceptibility to acute events.

**Figure 1:**
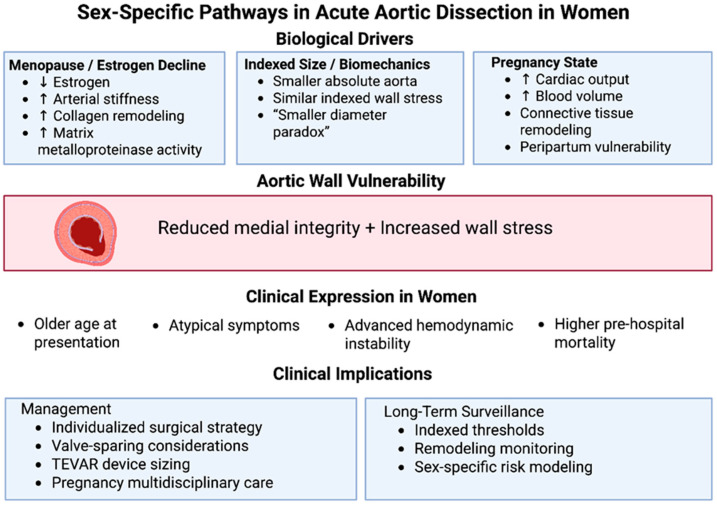
Sex-specific pathways in acute aortic dissection in women. Schematic representation of the biological and clinical mechanisms underlying sex-related differences in acute aortic dissection (AAD). Biological driver, including menopause-associated estrogen decline, indexed biomechanical stress related to smaller absolute aortic dimensions, and pregnancy-induced hemodynamic and connective tissue remodeling, converge to increase aortic wall vulnerability through reduced medial integrity and elevated wall stress. These mechanisms contribute to distinct clinical expression in women, characterized by older age at presentation, atypical symptom profiles, advanced hemodynamic instability, and higher pre-hospital mortality. Recognition of these pathways informs sex-specific management strategies, including individualized surgical planning, valve-sparing considerations, careful thoracic endovascular device sizing, multidisciplinary pregnancy care, and long-term surveillance incorporating indexed thresholds and sex-specific risk modeling.

Pregnancy provides a dynamic model of hormonally mediated vascular remodeling. Gestation is associated with a 30–50% increase in circulating blood volume and significant elevation in cardiac output, creating sustained hemodynamic stress on the aortic wall. Concurrently, progesterone, estrogen, and other pregnancy-related hormones promote connective tissue remodeling, including alterations in collagen structure and increased matrix metalloproteinase activity [10,11,12,13,14]. These changes, though physiologically adaptive, may transiently reduce medial integrity, particularly in women with underlying heritable thoracic aortic disease. Importantly, pregnancy-associated dissections may occur even in the absence of marked preexisting dilation, reinforcing the concept that acute hormonal and biomechanical shifts can precipitate dissection independently of absolute diameter criteria. The convergence of hormonal modulation indexed biomechanical stress, and pregnancy-related vascular remodeling provides a unifying framework for understanding the sex-specific clinical trajectory of acute aortic dissection in women (Figure 1). These biological and biomechanical differences do not remain confined to the cellular and molecular level, they manifest directly in the clinical presentation of women with acute aortic dissection, shaping symptom patterns, hemodynamic status at arrival, and the diagnostic challenges encountered in emergency settings.

Key sex-specific differences across epidemiology, biological mechanisms, clinical presentation, and early outcomes are summarized in Table 1.

Taken together, these mechanistic considerations suggest that sex differences in acute aortic dissection reflect an interplay between vascular aging, endocrine modulation, genetic susceptibility, and biomechanical stress. The convergence of menopausal hormonal withdrawal indexed aortic size considerations, and pregnancy-related connective tissue remodeling provides a biological framework that may explain clinical observations of later presentation, smaller dissection diameters, and distinct timing patterns in women. Incorporating these mechanistic insights into risk assessment models may ultimately refine surveillance strategies and guide more individualized thresholds for intervention.

## 5. Clinical Presentation and Diagnostic Challenges in Women

Clinical presentation of acute aortic dissection in women differs in several important respects from that observed in men, and these differences may contribute directly to delays in diagnosis and adverse outcomes. Data from large registries, including IRAD, consistently demonstrate that women present at older ages and are more likely to exhibit atypical or less specific symptoms at onset [2,3,4]. While abrupt chest pain remains the most common presenting symptom in both sexes, women are less likely to report the classic “tearing” or “ripping” quality of pain and may more frequently present with nonspecific complaints such as syncope, altered mental status, or signs of heart failure [2,3]. Neurological deficits, hypotension, and pericardial effusion or tamponade have also been reported more commonly in women at the time of presentation, reflecting either more advanced disease at diagnosis or potentially different patterns of dissection propagation [2,4]. These features complicate early recognition, particularly in emergency settings where atypical symptoms may initially prompt evaluation for alternative diagnoses.

Diagnostic delay represents a critical and recurring theme in the evaluation of women with AAD. Older age at presentation, the higher prevalence of comorbidities, and the absence of classic pain descriptors may lower clinical suspicion and prolong time to definitive imaging [2,3,4]. Furthermore, women appear less likely to have a prior diagnosis of known thoracic aortic aneurysm at the time of dissection, suggesting potential gaps in surveillance or earlier risk identification [2,3]. These factors collectively contribute to a higher proportion of women presenting in hemodynamically unstable states, including shock or cardiac tamponade, which are strongly associated with early mortality. Importantly, studies have also documented higher pre-hospital mortality rates among women, implying that a subset of patients may never reach surgical care [3,15]. The interplay between biological susceptibility, delayed recognition, and advanced presentation likely amplifies early risk.

Imaging strategies for suspected AAD are similar in women and men, with contrast-enhanced computed tomography remaining the primary diagnostic modality in most clinical settings. However, in pregnant patients or those in the peripartum period, imaging decisions require careful balancing of maternal and fetal considerations, and alternative modalities such as magnetic resonance imaging or transesophageal echocardiography may be preferentially utilized when feasible [10,11,12,13,14]. The need for heightened clinical vigilance in women, particularly older patients with atypical symptoms and younger patients during pregnancy or the postpartum period, is paramount. Recognition of these sex-specific presentation patterns is essential to reduce diagnostic delay, facilitate timely surgical referral, and ultimately improve survival outcomes.

## 6. Sex-Specific Considerations in Imaging and Diagnosis

Timely and accurate imaging is the cornerstone of AAD diagnosis, yet sex-specific factors may influence both the threshold for and interpretation of diagnostic imaging in women. CT angiography (CTA) of the aorta remains the primary diagnostic modality in suspected AAD, offering high sensitivity and specificity for dissection detection, flap characterization, and branch vessel involvement [19,20]. However, the clinical benefit of CTA is contingent on timely acquisition, and the higher prevalence of atypical or non-specific symptoms in women, including back pain, syncope, and generalized weakness in the absence of classic tearing chest pain, may reduce clinical suspicion and delay the threshold for definitive imaging [2,3]. Emergency providers should therefore maintain a lower threshold for aortic CTA in women presenting with acute chest or back pain in the presence of hypertension, older age, or known connective tissue disease, even when the symptom profile does not conform to classic descriptions.

Point-of-care ultrasound (POCUS) represents an increasingly valuable adjunct in the emergency evaluation of suspected AAD, particularly in hemodynamically unstable patients in whom immediate CTA may not be feasible. Focused cardiac ultrasound can rapidly identify pericardial effusion, aortic root dilation, and aortic regurgitation—findings that carry heightened relevance in women, who present more frequently with pericardial tamponade and hemodynamic compromise at the time of diagnosis [2,3,15]. While POCUS cannot replace definitive cross-sectional imaging, its integration into emergency triage protocols may facilitate earlier recognition of high-risk women and prompt expedited transfer to aortic surgery centers.

Imaging surveillance strategies for high-risk women warrant particular attention. Current ACC/AHA and ESC guidelines recommend serial aortic imaging in patients with known heritable thoracic aortic disease, prior aortic dilation, or a family history of aortic events, with surveillance intervals determined by the underlying diagnosis and rate of aortic growth [19,20]. In women, the consistent observation that dissection may occur at smaller absolute aortic diameters reinforces the importance of indexed measurements, including aortic size index and height-indexed diameter, in serial follow-up, rather than reliance on absolute thresholds alone [7,9]. Women with HTAD who are considering pregnancy require preconception imaging assessment and a structured multidisciplinary surveillance plan encompassing the third trimester and early postpartum period, when dissection risk is highest [10,11,13,19]. Integration of sex-specific indexed thresholds into surveillance protocols represents an important and currently underimplemented opportunity to improve early identification of women at risk.

## 7. Management Strategies and Therapeutic Considerations in Women

### 7.1. Type A Acute Aortic Dissection

Management of acute aortic dissection is dictated primarily by anatomical classification and hemodynamic status, with emergent surgical repair recommended for type A dissections and initial medical therapy for uncomplicated type B disease [21]. Recent ESC guidelines for peripheral arterial and aortic diseases reaffirm the urgency of surgical intervention in type A dissection and emphasize structured follow-up and individualized imaging surveillance in chronic phases [20]. However, sex-related differences in clinical presentation, age at diagnosis, and comorbidity burden inevitably influence therapeutic decision-making. Women with type A AAD present at older ages and more frequently with hemodynamic instability, tamponade, or neurological compromise, factors that significantly increase operative risk [2,3,4]. Consequently, several registry analyses have reported lower operative rates in women compared with men, particularly among the elderly, suggesting that perceived operative risk and frailty may influence decisions regarding surgical candidacy [2,3,4]. Even among women who undergo surgery, some cohorts have described less extensive aortic repair, with shorter cardiopulmonary bypass times and reduced frequency of complex arch reconstructions or adjunct cerebral perfusion strategies [15,17,22]. While these differences may reflect individualized risk assessment rather than overt bias, they raise important considerations regarding whether surgical aggressiveness should differ based solely on sex or age when long-term survival is at stake.

Operative strategy in type A dissection also intersects with anatomical and biological considerations. Women often dissect at smaller absolute aortic diameters [7,9], which challenges reliance on traditional size thresholds for prophylactic surgery and may suggest that some dissections occur in the absence of previously recognized high-risk dilation. In the acute setting, surgical goals remain excision of the primary intimal tear, prevention of rupture, and restoration of true lumen flow; however, decisions regarding root replacement, valve-sparing procedures, or more extensive arch intervention must account for patient age, tissue quality, and anticipated longevity. In younger women, particularly those of childbearing potential, valve-sparing root replacement may offer advantages by avoiding lifelong anticoagulation, whereas mechanical prostheses introduce future pregnancy-related risks that require careful counseling [10,11,12,13,14]. Conversely, in older women with advanced age and frailty, a more limited repair may reduce operative time and perioperative morbidity, though at the potential cost of increased late reintervention risk. These trade-offs underscore the importance of individualized surgical planning that integrates anatomical extent, physiological reserve, and long-term considerations.

### 7.2. Type B Acute Aortic Dissection

In type B acute aortic dissection (TBAD), initial management strategy is determined by the presence or absence of complications such as malperfusion, refractory pain, uncontrolled hypertension, rupture, or rapid aortic expansion. Optimal medical therapy with strict blood pressure and heart rate control remains the cornerstone for uncomplicated presentations, with the goal of reducing shear stress on the aortic wall and promoting false lumen stabilization. Observational registry data suggest that women are more frequently managed conservatively compared with men in certain cohorts [16,23,24]. Whether this reflects older age at presentation, higher comorbidity burden, anatomical considerations, or referral patterns remains uncertain, but it underscores potential differences in therapeutic thresholds.

When complications arise, thoracic endovascular aortic repair (TEVAR) has become the preferred intervention in many centers due to lower early morbidity compared with open repair. Contemporary analyses indicate broadly comparable short-term procedural success and in-hospital mortality between women and men undergoing TEVAR [25,26,27,28]. However, anatomical and biomechanical differences may influence procedural planning and technical execution. Women generally have smaller access vessels, including reduced iliofemoral artery diameter, which may increase the risk of access-related complications such as arterial injury or need for alternative access strategies. Careful pre-procedural imaging assessment and meticulous device selection are therefore particularly important in female patients to avoid oversizing, endoleak, or retrograde type A dissection.

The “smaller diameter paradox” may also have implications in TBAD management. Women frequently dissect at smaller absolute aortic diameters [7,9], raising questions regarding whether intervention thresholds for aneurysmal degeneration following TBAD should be indexed to body surface area rather than based solely on absolute size. Excessive device oversizing in smaller aortas may alter wall stress distribution and potentially influence remodeling dynamics. While current data do not demonstrate consistent sex-based differences in early TEVAR outcomes, long-term aortic remodeling patterns, including rates of false lumen thrombosis, true lumen expansion, distal aneurysmal progression, and reintervention, have not been sufficiently characterized in sex-stratified analyses.

Biological differences in extracellular matrix remodeling may further influence post-dissection aortic behavior. Hormonal status and vascular aging may alter collagen turnover and medial integrity, potentially affecting the stability of the dissected segment over time [7,8]. In older women, increased arterial stiffness and altered compliance may impact hemodynamic loading conditions and influence chronic remodeling trajectories. Whether these factors translate into clinically meaningful differences in long-term freedom from aneurysmal degeneration remains an area requiring further prospective investigation.

Long-term surveillance is therefore essential in women with TBAD, regardless of initial treatment strategy. Structured imaging follow-up is required to monitor false lumen status, aortic diameter progression, and device integrity following TEVAR. Given the limited availability of sex-stratified remodeling data, vigilance in long-term follow-up is particularly important. Additionally, risk factor optimization, including strict blood pressure control, remains critical in both medically and interventionally managed patients. Although beta-blocker therapy is standard in acute and chronic management, data specific to sex-related differences in pharmacologic response remain limited [16,23,24].

In summary, while short-term outcomes of TEVAR appear comparable between sexes, management of type B acute aortic dissection in women requires careful attention to anatomical considerations, device selection, and long-term remodeling surveillance. The integration of indexed diameter assessment and further study of sex-specific remodeling dynamics may help refine intervention thresholds and improve long-term outcomes.

### 7.3. Pregnancy-Associated Acute Aortic Dissection

Acute aortic dissection during pregnancy is rare but carries substantial maternal and fetal risk. According to the 2017 UK-MBRRACE report, aortic dissection ranked as the third leading cause of cardiovascular-related maternal mortality, highlighting its disproportionate clinical impact despite low absolute incidence [10]. The condition affects approximately 4–5 per million pregnancies [10], with cases clustering during the third trimester and early postpartum period, when hemodynamic stress and hormonal influences are most pronounced. Stanford type A dissections are more frequently observed antepartum, whereas type B dissections tend to occur in the postpartum period; notably, the mode of delivery does not appear to influence the occurrence of postpartum dissection [12].

The pathophysiology of pregnancy-associated dissection reflects a convergence of hemodynamic and hormonal factors. Pregnancy induces a marked increase in blood volume and cardiac output, accompanied by hormonally mediated connective tissue remodeling involving collagen turnover, elastin fragmentation, and matrix metalloproteinase activation [10,11,12,13,14]. These adaptive changes facilitate gestation but may transiently weaken the aortic media, particularly in women with underlying heritable thoracic aortic disease (HTAD). Importantly, dissections may occur even in the absence of marked preexisting dilation, underscoring the dynamic interaction between biomechanical stress and intrinsic aortic vulnerability.

Early diagnosis and coordinated intervention are critical to improving maternal and fetal outcomes. Management requires multidisciplinary collaboration among cardiothoracic surgery, maternal–fetal medicine, anesthesia, cardiology, and critical care teams [12]. ESC guidelines on cardiovascular disease during pregnancy strongly recommend preconception counseling, risk stratification based on aortic size and genetic background, and coordinated multidisciplinary management throughout gestation and the postpartum period [6,29]. In cases of Stanford type A dissection during pregnancy, standard management typically involves emergency delivery, often by cesarean section, followed by surgical repair of the aorta when gestational age permits. Maternal and fetal mortality have been shown to decrease when cesarean delivery is performed prior to aortic replacement after 28 weeks of gestation [12]. In earlier gestational stages, management must be individualized, balancing fetal viability with maternal survival.

Preventive strategies are particularly important in women with known or suspected HTAD. Genetic testing is recommended prior to pregnancy when diagnosis is uncertain, as risks vary across specific aortopathies [11]. Comprehensive imaging of the entire aorta using magnetic resonance angiography before conception is advised [11]. Women with significant aortic enlargement should receive detailed counseling regarding maternal risk, and prophylactic aortic surgery before pregnancy is generally recommended when diameter thresholds are met; in certain high-risk scenarios, pregnancy avoidance may be advised [11]. Surgical options such as the Bentall procedure and valve-sparing root replacement remain appropriate in women planning future pregnancies; however, the need for lifelong anticoagulation following mechanical valve implantation introduces additional complexities in anticoagulation management during gestation [10].

Delivery planning and postpartum surveillance are equally critical. Vaginal delivery with early epidural anesthesia may be appropriate in women with aortic root diameters under 40 mm and no additional risk factors, whereas cesarean section is recommended for those with diameters exceeding 45 mm or considered high risk [10]. Postpartum vulnerability persists beyond delivery, with the risk of dissection remaining elevated for up to six months [10]. ESC pregnancy recommendations highlight the early postpartum period as a continued high-risk window, supporting extended clinical vigilance and structured follow-up [6]. High-risk women should be monitored closely in the immediate postpartum period, ideally remaining hospitalized for at least one week after delivery. For those residing far from specialized centers, temporary proximity to a tertiary aortic surgery center for four to six weeks postpartum is advisable to ensure rapid access to emergency care if needed. Ongoing surveillance imaging and prompt evaluation of new symptoms are essential during this period.

Recent prospective data from the Registry of Pregnancy and Cardiac Disease (ROPAC) III, which followed pregnant women with known aortic disorders between 2018 and 2023, provide additional insights into postpartum management [13]. The study demonstrated no association between breastfeeding and postpartum aortic complications. Additionally, beta-blocker therapy was not associated with a reduction in major adverse cardiac events, aortic dissection, or aortic growth, although breastfeeding itself was associated with a lower incidence of major adverse cardiac events. These findings underscore the complexity of risk modification during and after pregnancy and highlight the need for individualized care strategies.

Collectively, pregnancy-associated acute aortic dissection represents a unique clinical entity characterized by dynamic hormonal influences, heightened hemodynamic stress, and distinct management considerations. Optimal outcomes depend on preconception counseling, structured surveillance, timely intervention, and coordinated multidisciplinary care extending well into the postpartum period. Beyond the unique context of pregnancy, sex-related differences in management extend across both type A and type B dissection, with distinct patterns of surgical decision-making, endovascular intervention, and outcomes that reflect the interplay of biological vulnerability, clinical presentation, and institutional factors.

## 8. Outcomes in Women with Aortic Dissection

### 8.1. Pre-Hospital and Early Mortality

Outcomes in women with acute aortic dissection are profoundly influenced by events occurring before definitive treatment is initiated. One of the most consistent findings across contemporary population-based and registry analyses is the higher rate of pre-hospital mortality among women compared with men [3,15]. This disparity suggests that a substantial proportion of women experience fatal complications prior to reaching specialized surgical care. Atypical symptom presentation, including less frequent reporting of classic tearing chest pain and higher rates of neurological symptoms or syncope, may reduce early clinical suspicion and delay emergent imaging [2,3,4]. Additionally, older age at presentation and greater comorbidity burden may contribute to diagnostic uncertainty, particularly in emergency settings where alternative diagnoses such as acute coronary syndrome or heart failure are initially considered. These factors collectively increase the likelihood of delayed recognition and hemodynamic deterioration.

Among patients who survive to hospital admission, women frequently present with more advanced disease states, including hypotension, cardiac tamponade, shock, and pericardial effusion, all of which are established predictors of early mortality [2,3,4]. Hemodynamic instability at presentation is not merely a marker of severity but may reflect delayed diagnosis, more aggressive propagation patterns, or differences in vascular compliance and medial integrity. Older age further compounds operative risk, as women presenting with type A dissection are often significantly older than their male counterparts [2,3,4]. Advanced age is associated with increased frailty, diminished physiologic reserve, and higher susceptibility to perioperative complications such as stroke, renal failure, and prolonged mechanical ventilation.

Historical registry data demonstrated higher in-hospital operative mortality among women undergoing surgical repair for type A dissection [2,3,4]. However, more recent analyses suggest that this mortality gap has narrowed, likely reflecting improvements in surgical technique, cerebral protection strategies, anesthesia management, and centralized care at high-volume aortic centers [4,15,17]. These improvements highlight the modifiable nature of some early disparities and suggest that optimized systems of care can mitigate sex-related risk differences. Nevertheless, certain cohorts continue to report higher early mortality in women, particularly among elderly subgroups [15,22], indicating that age-adjusted risk remains relevant.

Another contributing factor may be differences in operative strategy. Some studies have reported lower rates of extensive aortic repair or complex arch reconstruction in women [15,17,22]. While shorter operative times may reduce immediate surgical stress, limited repair strategies could potentially influence long-term outcomes if residual disease remains. It remains unclear whether these differences represent appropriate tailoring of surgical aggressiveness to age and frailty or reflect subconscious bias in therapeutic decision-making. Further investigation into sex-adjusted operative risk modeling may clarify whether treatment intensity is optimally calibrated.

In type B acute aortic dissection, early mortality appears less dramatically different between sexes, particularly when medical therapy is appropriately administered [16,23]. However, higher age and comorbidity burden in women may influence short-term outcomes in complicated presentations. When thoracic endovascular aortic repair is performed for complicated TBAD, contemporary studies generally report comparable early mortality between sexes [25,26,27,28], suggesting that procedural standardization may attenuate early disparities in this subgroup.

Overall, pre-hospital mortality and early in-hospital outcomes in women reflect a convergence of biological vulnerability, atypical clinical presentation, older age, and potential delays in diagnosis and referral. The narrowing of early mortality gaps in contemporary practice is encouraging, but persistent disparities in certain subgroups underscore the need for heightened clinical vigilance, rapid imaging pathways, and centralized management in experienced aortic centers. Early recognition and timely intervention remain the most powerful modifiable determinants of survival in women with acute aortic dissection.

### 8.2. Long-Term Outcomes and Aortic Remodeling

While improvements in perioperative care have narrowed early mortality differences in acute aortic dissection, long-term outcomes remain less clearly defined and may reveal persistent sex-related disparities. Several analyses suggest that women experience less favorable long-term survival following repair of type A dissection in certain cohorts [15,18]. Although age at presentation likely contributes to this observation, it does not fully explain potential differences in late mortality. Residual aortic disease burden, chronic false lumen patency, progressive aneurysmal degeneration, and comorbidity accumulation may all influence long-term trajectories. Importantly, women are less likely to have had a prior diagnosis of thoracic aortic aneurysm at the time of acute presentation [2,3], raising the possibility that surveillance gaps persist even after initial repair.

Aortic remodeling following dissection represents a critical determinant of late outcomes. Successful remodeling is characterized by false lumen thrombosis, true lumen expansion, and stabilization or reduction in aortic diameter. However, persistent false lumen patency is associated with progressive dilation and increased risk of reintervention. Sex-stratified data on remodeling dynamics remain limited, but biological differences in extracellular matrix composition and vascular stiffness may influence chronic remodeling patterns [7,8]. The decline in estrogen following menopause is associated with increased arterial stiffness and altered collagen turnover [7,8], potentially affecting the mechanical behavior of the chronically dissected aorta. Whether these factors translate into differential rates of distal aneurysmal degeneration or need for late reintervention in women remains incompletely characterized.

In type B dissection, long-term outcomes are closely tied to false lumen behavior and the adequacy of initial management. Women treated medically for uncomplicated TBAD appear to have comparable short-term survival to men [16,23], yet long-term data stratified by sex remain sparse. Following thoracic endovascular aortic repair, early mortality and procedural success are generally similar between sexes [25,26,27,28]; however, long-term durability, rates of distal aortic expansion, and need for secondary intervention have not been consistently evaluated in sex-specific analyses. Anatomical factors such as smaller baseline aortic diameter and smaller access vessels in women may influence device sizing and hemodynamic remodeling, potentially affecting late outcomes.

The “smaller diameter paradox” further complicates interpretation of long-term risk. Women frequently dissect at smaller absolute diameters [7,9], which may imply that diameter-based thresholds for reintervention in the chronic phase require reassessment when indexed to body surface area. Absolute size progression may underestimate relative wall stress in smaller individuals, particularly in the presence of persistent hypertension or increased arterial stiffness. Prospective validation of indexed follow-up criteria may therefore be warranted to refine surveillance strategies in women.

Reintervention rates represent another important dimension of long-term outcomes. Although existing datasets do not consistently demonstrate higher reintervention rates in women, underreporting and limited follow-up duration in registries may obscure subtle differences. Furthermore, older age at initial presentation in women may influence candidacy for secondary procedures, potentially affecting long-term survival independently of biological remodeling patterns.

Structured lifelong surveillance is essential for all patients with a history of aortic dissection, but heightened vigilance may be particularly important in women given the confluence of vascular aging, hormonal influences, and potential indexing considerations. ESC recommendations underscore the importance of standardized long-term imaging protocols and risk-factor optimization to mitigate progressive aortic remodeling [20]. Imaging intervals should be individualized based on aortic size, growth rate, false lumen status, and comorbidity burden. In women with heritable thoracic aortic disease or prior pregnancy-associated dissection, long-term monitoring is especially critical due to potential recurrent risk [10,11,12,13,14].

In summary, while early mortality disparities between sexes appear to be narrowing, long-term outcomes and aortic remodeling patterns in women remain insufficiently characterized. Differences in vascular biology, indexed aortic dimensions, and age-related stiffness may influence chronic dissection behavior in ways not fully captured by existing registries. Dedicated sex-stratified longitudinal studies are needed to clarify remodeling trajectories, optimize surveillance protocols, and refine reintervention thresholds in women with acute aortic dissection.

### 8.3. Pregnancy-Associated Outcomes

Pregnancy-associated acute aortic dissection represents a rare but particularly high-risk clinical entity in which maternal and fetal outcomes are closely intertwined. Although the overall incidence remains low (approximately 4–5 cases per million pregnancies [10]) its contribution to maternal cardiovascular mortality is disproportionate, ranking among the leading causes of pregnancy-related cardiac death in national reports such as UK-MBRRACE [10]. Maternal mortality is strongly influenced by the timing of diagnosis, hemodynamic stability at presentation, and the rapidity with which definitive surgical care is delivered. Delayed recognition, particularly in women presenting with nonspecific symptoms such as chest discomfort, dyspnea, or neurological manifestations, may result in catastrophic deterioration before intervention can be undertaken.

Outcomes differ according to dissection type and gestational age. Stanford type A dissections, which more commonly occur during pregnancy prior to delivery, carry substantial maternal risk if not treated emergently [12]. When gestational age exceeds 28 weeks, maternal and fetal mortality have been shown to decrease when cesarean delivery is performed immediately prior to aortic repair [12]. In earlier gestational stages, management decisions become more complex, often prioritizing maternal survival while recognizing that fetal viability may be limited. Stanford type B dissections are more frequently observed in the postpartum period [12], a time characterized by abrupt hemodynamic shifts and persistent hormonal effects on connective tissue remodeling. Importantly, the mode of delivery does not appear to independently influence the risk of postpartum dissection [12], suggesting that intrinsic vascular vulnerability rather than obstetric technique is the principal driver.

Fetal outcomes are highly dependent on maternal stability and gestational age at the time of intervention. Early gestational dissections are associated with higher rates of fetal loss, whereas dissections occurring after fetal viability may allow for coordinated delivery and surgical repair with improved neonatal survival [12]. Multidisciplinary planning is therefore essential to optimize both maternal and fetal outcomes, particularly in tertiary centers with expertise in complex aortic surgery and high-risk obstetrics [10,11,12,13,14].

Postpartum outcomes warrant particular attention, as the risk of dissection remains elevated for up to six months following delivery [10]. Hemodynamic normalization does not immediately reverse pregnancy-associated connective tissue changes, and the early postpartum period may be characterized by ongoing vascular vulnerability. For this reason, high-risk women should undergo close inpatient monitoring for at least one week following delivery, with extended proximity to specialized aortic centers recommended when geographic access to emergency care is limited [10]. Structured surveillance imaging and prompt evaluation of new symptoms are critical during this period.

Long-term outcomes in women with pregnancy-associated dissection depend on underlying aortic pathology. Women with heritable thoracic aortic disease require lifelong surveillance, as future pregnancies may carry recurrent risk. Data from the Registry of Pregnancy and Cardiac Disease (ROPAC) III provide additional insights into postpartum risk modification, demonstrating no association between breastfeeding and increased aortic complications, and no significant reduction in major adverse cardiac events or aortic growth with beta-blocker therapy in this cohort [13]. Interestingly, breastfeeding was associated with a lower incidence of major adverse cardiac events, though the mechanisms underlying this observation remain uncertain and warrant further investigation.

Collectively, pregnancy-associated acute aortic dissection highlights the dynamic interaction between hormonal modulation, hemodynamic stress, and underlying aortopathy. Maternal survival depends critically on rapid recognition and coordinated multidisciplinary management, while fetal outcomes are closely linked to gestational age and maternal stability. Extended postpartum surveillance and individualized long-term follow-up strategies are essential to mitigate recurrent risk and optimize long-term cardiovascular health in this uniquely vulnerable population.

A consolidated overview of sex-related differences in therapeutic decision-making and long-term surveillance is presented in Table 2.

## 9. Discussion

The findings synthesized in this review converge on a central clinical message: women with acute aortic dissection represent a biologically and clinically distinct population whose risk is inadequately captured by diagnostic and therapeutic frameworks derived predominantly from male cohorts. At the core of this disparity lies the smaller diameter paradox—the consistent observation that women dissect at smaller absolute aortic diameters—which, when viewed through the lens of indexed biomechanical stress, hormonal vascular aging, and pregnancy-related remodeling, reflects a fundamentally different trajectory of aortic vulnerability [7,9,19]. The sections that follow address how this recognition should inform risk stratification, management decisions, and future research priorities.

Sex-related differences in AAD extend beyond epidemiologic observation and instead reflect a complex interplay of biological, clinical, and systemic factors. Women consistently present at older ages and are more likely to exhibit atypical or less specific symptoms compared with men, patterns that may contribute to diagnostic delay and advanced hemodynamic compromise at presentation [2,3,4]. Higher rates of pre-hospital mortality among women further suggest that disparities begin before definitive surgical intervention is initiated [3,15].

In addition to biological determinants, structural and diagnostic factors likely contribute to these disparities. Women are less frequently diagnosed with thoracic aortic aneurysm prior to dissection and experience higher rates of pre-hospital mortality, suggesting gaps in surveillance and early recognition [2,3]. Atypical symptom patterns may increase the likelihood of misclassification as alternative acute cardiovascular conditions, thereby delaying definitive imaging. Furthermore, risk stratification algorithms and diagnostic thresholds have historically been derived from male-predominant cohorts, which may inadvertently reinforce under-recognition in women. Similar patterns of delayed diagnosis and risk underestimation have been observed across the broader spectrum of arterial vascular disease, as highlighted in recent American Heart Association scientific statements [5]. Addressing these disparities requires not only biological insight but also refinement of emergency imaging pathways, structured surveillance strategies, and heightened clinical vigilance.

The key sex-specific clinical implications derived from contemporary guidelines are summarized in Table 3.

Hormonal and vascular aging mechanisms appear central to understanding sex-specific disease expression. Estrogen exerts protective vascular effects through modulation of endothelial nitric oxide production, anti-inflammatory pathways, and regulation of extracellular matrix turnover. The decline in estrogen following menopause has been associated with increased arterial stiffness and alterations in collagen–elastin balance within the aortic wall, processes that may predispose to medial degeneration and dissection [7,8]. These mechanisms may help explain both the later age of presentation in women and the observation that dissections frequently occur at smaller absolute aortic diameters [7,9]. The “smaller diameter paradox” challenges reliance on fixed surgical thresholds based solely on absolute size and supports the consideration of indexed measurements in risk stratification. Current ACC/AHA recommendations acknowledge the limitations of absolute diameter thresholds and support individualized decision-making incorporating indexed aortic dimensions and clinical context [19] Prospective evaluation of sex-specific thresholds is warranted to determine whether current guidelines adequately capture risk in women.

Pregnancy further illustrates the interaction between hormonal milieu and biomechanical stress. Gestation is characterized by substantial increases in circulating blood volume and cardiac output, accompanied by hormonally mediated connective tissue remodeling and increased matrix metalloproteinase activity [10,11,12,13,14]. These adaptive changes may transiently weaken the aortic media, particularly in women with underlying heritable thoracic aortic disease. The clustering of dissections during the third trimester and early postpartum period reinforces the concept that dynamic hormonal shifts and acute hemodynamic stress act synergistically to precipitate dissection [10,11,12]. Importantly, pregnancy-associated dissections may occur in the absence of marked preexisting dilation, highlighting the limitations of diameter-based risk assessment alone.

Management patterns also reflect the intersection of biological and clinical factors. Women with type A dissection are often older and present with higher rates of hypotension, tamponade, or shock, variables strongly associated with operative mortality [2,3,4]. Although earlier registry data suggested higher surgical mortality in women, more contemporary analyses indicate that this gap has narrowed with advances in perioperative care and centralization of surgical services [4,15,17]. Nevertheless, some cohorts report lower operative rates and less extensive surgical repair in women with type A AAD [2,3,4,15,22], raising important questions regarding risk perception, referral patterns, and long-term implications of limited repair strategies. In type B dissection, observational data suggest that women are more frequently managed conservatively [16,23,24], though short-term outcomes following thoracic endovascular aortic repair appear comparable between sexes in contemporary series [10,26,27,28]. Long-term remodeling patterns and reintervention rates following type B management, however, remain insufficiently characterized in sex-stratified analyses.

System-level factors likely contribute to observed disparities. Women are less likely to have a prior diagnosis of thoracic aortic aneurysm at the time of dissection [2,3], suggesting potential gaps in surveillance or earlier risk identification. Atypical symptom profiles may reduce clinical suspicion in emergency settings, leading to delayed imaging and treatment. Addressing these gaps requires heightened awareness, integration of sex-specific risk considerations into diagnostic algorithms, and rapid referral pathways to experienced aortic centers. Furthermore, long-term survival after repair has been reported as less favorable in women in certain analyses [15,18], though the underlying drivers, whether biological, procedural, or related to comorbidity burden, remain incompletely defined.

Sex-related differences in acute aortic dissection must also be understood within the broader framework of social determinants of cardiovascular health. As highlighted by Costa et al., sex and gender function not only as biological variables but as key social determinants that shape vascular disease risk, recognition, and outcomes through differential access to healthcare, health literacy, socioeconomic position, and systemic gender biases embedded in clinical practice [30]. In the context of AAD, these structural factors may compound biologically mediated disparities: women presenting to non-specialist centers, those with limited access to advanced imaging, or those whose atypical symptom profiles trigger anchoring bias in emergency providers may face compounded diagnostic disadvantage that is not captured in registry data alone. A gender-sensitive approach to cardiovascular care—one that integrates biological sex with social and structural context—is therefore essential to fully address the outcome disparities documented in this review [5,6,30].

Several methodological limitations of the existing literature merit explicit acknowledgment. The observational and registry-based nature of most available data precludes causal inference, and heterogeneity in outcome definitions, follow-up duration, and center volume across studies limits direct comparability. Women remain underrepresented in many surgical and endovascular registries, reducing statistical power for robust sex-stratified subgroup analyses [2,4,15,17]. Critically, few studies apply multivariable models with sex-specific adjustment or test for interaction between sex and key covariates such as age, aortic diameter, and comorbidity burden, raising the possibility that reported sex differences are confounded by the consistently older age and greater comorbidity load in women [2,3,15]. The absence of prospective, sex-stratified trial data in acute aortic dissection represents a fundamental gap that limits the strength of evidence underlying current guideline recommendations [19,20].

Several knowledge gaps merit focused investigation. Women remain underrepresented in many surgical and endovascular registries, limiting statistical power for robust sex-stratified analyses. Current guideline recommendations for prophylactic aortic surgery rely largely on absolute diameter criteria derived from predominantly male populations; prospective validation of indexed dimensions and sex-specific predictive models is needed [7,9]. Translational studies exploring sex-based differences in extracellular matrix remodeling, inflammatory signaling, and vascular aging may further clarify mechanistic pathways [7,8]. Finally, pregnancy-associated aortic disease requires standardized international registries and consensus management strategies to optimize maternal and fetal outcomes [10,11,12,13,14].

An important future direction involves the development of sex-specific risk prediction models for acute aortic dissection. Current risk stratification strategies and surgical thresholds are largely derived from cohorts in which men predominate, and most rely heavily on absolute aortic diameter as the principal determinant of intervention [7,9]. However, the consistent observation that women dissect at smaller absolute diameters, coupled with differences in body surface area, vascular stiffness, menopausal status, and pregnancy-related risk, suggests that traditional models may inadequately capture individualized risk in female patients. Incorporating indexed aortic dimensions, hormonal status, reproductive history, and potentially sex-specific biomarkers of extracellular matrix remodeling into predictive algorithms may improve identification of high-risk women before catastrophic events occur. Prospective validation of such models, including sex-stratified performance analysis, will be essential to determine whether tailored risk assessment can reduce diagnostic delay, optimize timing of prophylactic surgery, and ultimately mitigate outcome disparities in women with thoracic aortic disease.

In summary, acute aortic dissection in women reflects a convergence of hormonal influences, vascular aging, genetic susceptibility, clinical presentation patterns, and healthcare system factors. While advances in surgical and endovascular management have improved early survival, disparities persist in diagnostic timing, pre-hospital mortality, and potentially long-term outcomes. A deliberate integration of sex as a biological and clinical variable in research, surveillance, and guideline development is essential to refine risk stratification and improve outcomes for women affected by this life-threatening condition.

## 10. Conclusions

Acute aortic dissection in women represents a complex clinical entity shaped by biological, hormonal, and systemic factors that extend beyond traditional epidemiologic differences. Women present at older ages, more frequently exhibit atypical symptoms, and are more likely to arrive in advanced hemodynamic states, contributing to higher pre-hospital mortality and early risk. Hormonal influences, menopause-associated vascular aging, and pregnancy-related connective tissue remodeling may alter aortic wall integrity and susceptibility to dissection, while the occurrence of events at smaller absolute diameters challenges reliance on fixed size thresholds alone. Although contemporary surgical advances have narrowed some in-hospital mortality differences in type A AAD, and endovascular outcomes appear broadly comparable between sexes in type B disease, important gaps remain in risk stratification, long-term outcome assessment, and sex-specific guideline development. Greater incorporation of indexed aortic dimensions, heightened clinical vigilance for atypical presentations, and multidisciplinary management pathways, particularly in pregnancy, are essential to optimize outcomes. Future research should prioritize sex-stratified analyses and mechanistic investigation of vascular remodeling to refine surveillance strategies and therapeutic thresholds. A more deliberate integration of sex-specific considerations into clinical practice and research may ultimately improve survival and long-term health for women affected by acute aortic dissection. Methodological advances, including prospective sex-stratified registries, standardized outcome definitions, and multivariable analyses incorporating sex-specific interaction terms, are prerequisites for generating the evidence needed to close these gaps.

## Figures and Tables

**Table 1:** Sex-Specific Characteristics in Acute Aortic Dissection. The Table summarizes key sex-specific differences in acute aortic dissection across epidemiology, biological mechanisms, clinical presentation, and early outcomes. Women demonstrate later age at presentation, dissection at smaller absolute diameters, and higher rates of atypical symptoms and pre-hospital mortality. Hormonal influences and pregnancy-related vascular remodeling contribute to unique pathophysiological considerations.

Domain	Observations in Women	Clinical Implications	Key References
Epidemiology	Older age at presentation; ~30–35% of cases; less prior aneurysm diagnosis	Possible under-recognition and delayed surveillance	[2,3,4]
Risk Profile	Hypertension prevalent; pregnancy and HTAD important modifiers	Need for sex-aware risk stratification	[10,11,12,13,14]
Aortic Dimensions	Dissection at smaller absolute diameters (“smaller diameter paradox”)	Consider indexed diameter thresholds	[7,9]
Hormonal Biology	Menopause-associated vascular stiffening; estrogen-related ECM regulation	Hormonal influence on medial integrity	[7,8]
Pregnancy Physiology	Increased cardiac output and connective tissue remodeling	Dynamic biomechanical stress; peripartum vulnerability	[10,11,12,13,14]
Clinical Presentation	More atypical symptoms; higher rates of tamponade, hypotension	Diagnostic delay; advanced presentation	[2,3,4]
Pre-Hospital Mortality	Higher than men	Early recognition crucial	[3,15]
Operative Rates (Type A)	Lower operative frequency in elderly women; less extensive repair in some cohorts	Possible therapeutic conservatism	[2,3,4,15,17,22]
Early Mortality Trends	Historical gap narrowing in contemporary practice	Centralization improves outcomes	[4,15,17]

**Table 2:** Sex-Specific Considerations in Management and Long-Term Outcomes. The Table summarizes sex-specific considerations in management strategies and long-term outcomes of acute aortic dissection. Although early outcomes after surgical and endovascular intervention are increasingly comparable between sexes, important differences persist in operative candidacy, pregnancy-related risk, and long-term aortic remodelling dynamics.

Clinical Scenario	Findings in Women	Implications for Management	Key References
Type A AAD	Older age; higher instability at presentation; less extensive repair in some series	Individualized surgical aggressiveness; frailty assessment	[2,3,4,15,17,22]
Valve Strategy	Pregnancy considerations relevant in younger women	Favor valve-sparing when feasible	[10,11,12,13,14]
Type B AAD (Medical)	More frequent conservative management	Strict BP control; close surveillance	[16,23,24]
TEVAR (Complicated TBAD)	Comparable short-term outcomes; smaller access vessels	Careful device sizing; avoid oversizing	[25,26,27,28]
Aortic Remodeling	Potential influence of vascular stiffness and ECM biology	Indexed follow-up thresholds; sex-stratified surveillance	[7,8]
Reintervention Risk	Data limited; possibly influenced by smaller baseline diameters	Need for prospective sex-stratified studies	[8,9,18]
Pregnancy-Associated AAD	High maternal risk; postpartum vulnerability up to 6 months	Multidisciplinary care; extended surveillance	[10,11,12,13]

**Table 3:** Sex-specific guidelines implications in aortic dissection.

Domain	Guideline/Evidence Anchor	Guideline-Based Considerations in AAD	Clinical Implication in Women
Pre-dissection risk assessment	ACC/AHA Aortic Disease Guideline 2022	Risk assessment should incorporate aortic size, growth rate, body size, and HTAD context; indexing concepts are emphasized for smaller individuals and genetic disease.	Women may be at risk at smaller absolute diameters; consider indexed size + growth trajectory when discussing “risk” and prophylactic strategies.
Smaller diameter paradox	Evidence/registries (IRAD + cohorts) + framed within ACC/AHA 2022 risk concepts	Observational data show women may present with type A AAD at smaller diameters and with later diagnosis; guidelines acknowledge individualized thresholds (esp. in HTAD/body size) but do not provide a sex-specific cut-off.	Avoid false reassurance based on diameter alone; “lower threshold to worry” in women with additional risk modifiers (growth, HTAD, pregnancy, uncontrolled HTN).
Pregnancy & postpartum high-risk window	ESC Pregnancy Guideline 2018 (and ESC Pregnancy 2025 update) + ACC/AHA 2022	Dissection risk increases in late pregnancy and early postpartum (notably first 6–12 weeks); preconception counselling and multidisciplinary care are recommended in known aortopathy/HTAD; imaging surveillance is advised in high-risk patients.	In women with known aortopathy/HTAD or prior dilation: structured preconception planning, serial imaging when indicated, and explicit postpartum follow-up plan.
Heritable thoracic aortic disease (HTAD)	ACC/AHA 2022 + ESC Aortic 2014/ESC PAAD 2024	HTAD syndromes and family history lower thresholds for intervention and intensify surveillance; pregnancy planning is part of risk management in women with HTAD.	Earlier prophylactic consideration and closer imaging surveillance—particularly for women considering pregnancy or with prior pregnancy complications.
Symptom recognition & diagnostic delay	ESC Aortic 2014/ESC PAAD 2024	AAD diagnosis requires urgent imaging based on clinical suspicion; women more often have atypical presentations and delayed recognition (evidence-driven).	Maintain lower threshold for CT/TEE in women with chest/back pain + risk factors; avoid anchoring on “non-classic” symptom pattern.
Operative risk & outcomes in type A AAD	ESC Aortic 2014/ESC PAAD 2024	Guidelines support emergent surgery for type A AAD; registry data show women have higher operative mortality partly due to older age and later presentation.	Emphasize systems-of-care: early recognition, rapid transfer, and Heart-Team pathways may reduce the sex gap in outcomes.

## Data Availability

No new data were created or analyzed in this study.

## Abbreviations

The following abbreviations are used in this manuscript:

AAD	Acute aortic dissection
BSA	Body Surface Area
BMI	Body mass index
HTAD	Heritable thoracic aortic disease
IRAD	International Registry of Acute Aortic Dissection
ICU	Intensive care unit
TAAD	Type A acute aortic dissection
MBBRACE-UK	Mothers and Babies: Reducing Risk through Audits and Confidential Enquiries across the UK
TBAD	Type B aortic dissections
COPD	Chronic obstructive pulmonary disease
TEVAR	Thoracic endovascular aortic repair
MACE	Major adverse cardiac events
NRD	Nationwide Readmissions Database
ROPAC	Registry of Pregnancy and Cardiac Disease
